# Protective effect of hydroxychloroquine on infections in patients with systemic lupus erythematosus: an observational study using the LUNA registry

**DOI:** 10.3389/fimmu.2023.1227403

**Published:** 2023-09-01

**Authors:** Chiharu Hidekawa, Ryusuke Yoshimi, Yusuke Saigusa, Jun Tamura, Noriko Kojitani, Naoki Suzuki, Natsuki Sakurai, Yuji Yoshioka, Yumiko Sugiyama-Kawahara, Yosuke Kunishita, Daiga Kishimoto, Kana Higashitani, Yuichiro Sato, Takaaki Komiya, Hideto Nagai, Naoki Hamada, Ayaka Maeda, Naomi Tsuchida, Lisa Hirahara, Yutaro Soejima, Kaoru Takase-Minegishi, Yohei Kirino, Nobuyuki Yajima, Ken-ei Sada, Yoshia Miyawaki, Kunihiro Ichinose, Shigeru Ohno, Hiroshi Kajiyama, Shuzo Sato, Yasuhiro Shimojima, Michio Fujiwara, Hideaki Nakajima

**Affiliations:** ^1^ Department of Stem Cell and Immune Regulation, Yokohama City University Graduate School of Medicine, Yokohama, Japan; ^2^ Clinical Laboratory Department, Yokohama City University Hospital, Yokohama, Japan; ^3^ Department of Biostatistics, Yokohama City University School of Medicine, Yokohama, Japan; ^4^ Center for Rheumatic Diseases, Yokohama City University Medical Center, Yokohama, Japan; ^5^ Division of Rheumatology, Department of Medicine, Showa University School of Medicine, Tokyo, Japan; ^6^ Department of Clinical Epidemiology, Kochi Medical School, Kochi University, Nankoku, Japan; ^7^ Department of Nephrology, Rheumatology, Endocrinology and Metabolism, Okayama University Graduate School of Medicine, Dentistry and Pharmaceutical Sciences, Okayama, Japan; ^8^ Department of Immunology and Rheumatology, Division of Advanced Preventive Medical Sciences, Nagasaki University Graduate School of Biomedical Sciences, Nagasaki, Japan; ^9^ Department of Rheumatology and Applied Immunology, Faculty of Medicine, Saitama Medical University, Morohongo, Japan; ^10^ Department of Rheumatology, Fukushima Medical University School of Medicine, Fukushima, Japan; ^11^ Department of Medicine (Neurology and Rheumatology), Shinshu University School of Medicine, Matsumoto, Japan; ^12^ Department of Rheumatology, Yokohama Rosai Hospital, Yokohama, Japan

**Keywords:** systemic lupus erythematosus (SLE), hydroxychloroquine, infection, Asian, multicenter registry

## Abstract

**Objectives:**

Infection is a leading cause of death in patients with systemic lupus erythematosus (SLE). Alt hough hydroxychloroquine (HCQ) has been reported to inhibit infection, evidence from Asian populations remains insufficient. We investigated this effect in Japanese SLE patients.

**Methods:**

Data from the Lupus Registry of Nationwide Institutions were used in this study. The patients were ≥20 years old and met the American College of Rheumatology (ACR) classification criteria revised in 1997. We defined “severe infections” as those requiring hospitalization. We analyzed the HCQ’s effect on infection suppression using a generalized estimating equation (GEE) logistic regression model as the primary endpoint and performed a survival analysis for the duration until the first severe infection.

**Results:**

Data from 925 patients were used (median age, 45 [interquartile range 35–57] years; female, 88.1%). GEE analysis revealed that severe infections were significantly associated with glucocorticoid dose (odds ratio [OR] 1.968 [95% confidence interval, 1.379–2.810], *p*<0.001), immunosuppressants (OR 1.561 [1.025–2.380], *p*=0.038), and baseline age (OR 1.043 [1.027–1.060], *p*<0.001). HCQ tended to suppress severe infections, although not significantly (OR 0.590 [0.329–1.058], *p*=0.077). Survival time analysis revealed a lower incidence of severe infections in the HCQ group than in the non-HCQ group (*p*<0.001). In a Cox proportional hazards model, baseline age (hazard ratio [HR] 1.029 [1.009–1.050], *p*=0.005) and HCQ (HR 0.322 [0.142–0.728], *p*=0.006) were significantly related to incidence.

**Conclusion:**

HCQ may help extend the time until the occurrence of infection complications and tends to decrease infection rates.

## Introduction

Systemic lupus erythematosus (SLE) is a systematic autoimmune disease characterized by inflammatory organ lesions secondary to autoantibody production and tissue deposition of immune complexes. Traditionally, in addition to glucocorticoids, immunosuppressive agents such as cyclophosphamide, azathioprine, and cyclosporine have been used for SLE worldwide for more than 30 years (1). In the 2000s, rituximab was introduced, and more recently, novel agents such as belimumab, anifrolumab, and voclosporin have emerged, leading to a decrease in the use of glucocorticoids and improvement in the clinical course of the disease. However, although the efficacy of SLE therapy has improved, infection remains one of the most important prognostic factors. Some reports suggest that infections are the cause of death in 31.7 to 36.5% of SLE patients (2, 3). Thus, prevention of infectious diseases is a critical element of SLE treatment.

Hydroxychloroquine (HCQ), an antimalarial drug, is used to treat patients with SLE worldwide. Many studies have reported that it can effect a reduction in the risk of organ damage and death (4–6); it has been suggested that it should be administered to all SLE patients (7). In addition to improving disease control, the drug may prevent infection and thrombosis (4). Despite multiple reports suggesting that HCQ is effective in controlling infectious diseases, no definite conclusions have been reached (8–11). Particularly in East Asia, data are limited on the infectious disease suppression effects of HCQ, and studies on large populations are required.

The Lupus Registry of Nationwide Institutions (LUNA) is the first multicenter registry of patients with SLE in Japan, including clinical data from 2016. The number of participating facilities has been gradually increasing, including 18 rheumatology departments of universities and general hospitals by 2021, with more than 1,000 patients enrolled in the registry. In this study, we evaluated the efficacy of HCQ in preventing infections in patients with SLE living in Japan, using data from the LUNA registry.

## Materials and methods

### Patient selection

The variables, definitions, and collection processes in the registry have been described in previous studies using LUNA data (12–14). Briefly, patients aged ≥ 20 years who met the 1997 ACR revised classification criteria for SLE (15) were included in the LUNA registry. We collected clinical data, including laboratory test results, disease activity, and treatment of patients enrolled in the registry, on an annual basis. The treatment of each patient was determined by the attending physician. In this study, we used data from patients who were enrolled in 2016 and followed up for 1–5 years until 2021. Data from patients whose HCQ medication status was known for less than one year were excluded.

### Outcome measures

This study aimed to determine whether HCQ used for SLE is also effective in reducing the incidence of infectious diseases. To this end, we first investigated the effect of HCQ on severe infection as the primary outcome. Patient data were obtained as person-years of follow-up and divided into two groups according to HCQ medication status at the one-year interval observation points. The HCQ group included patients taking HCQ at both observation points; the non-HCQ group included those not taking HCQ at either observation point. If HCQ medication status differed before and after the one-year period, the patient was excluded from the analysis.

As a secondary outcome, we analyzed the effect of HCQ medication on the time to the first severe infection during the observation period. For survival analysis, we allocated patients who received HCQ for not less than one year during the observation period into the HCQ group, and those who did not into the non-HCQ group. For the HCQ group, data at enrollment were used as baseline data, and data immediately after starting HCQ were used for patients who started taking HCQ during follow-up. If HCQ was discontinued, patients were censored at the last follow-up. For the non-HCQ group, data at enrollment were used as baseline data.

### Definition of severe infections

We defined “severe infection” as “infection requiring inpatient care and condition monitoring” in this study. The following types of infections were collected independently: pulmonary, urinary tract, skin and soft tissue, intra-abdominal, gastroenteritis, sepsis, infectious arthritis, and upper respiratory tract, and an “others” category.

### Statistical analysis

Characteristics, rates, and proportions were used for categorical data, whereas the median and range were used for continuous data. The Mann–Whitney *U* test was used in the analysis of continuous variables since nonparametric data were observed. The chi-squared test was used for categorical variables. The collected data included: demographic factors such as sex and age; blood laboratory data; the SELENA-SLE Disease Activity Index (SLEDAI); the Systemic Lupus International Collaborating Clinics/American College of Rheumatology Damage Index (SDI); and the medication status of steroids, immunosuppressives (cyclophosphamide, mycophenolate mofetil, mizoribine, methotrexate, azathioprine, tacrolimus, cyclosporine, rituximab, and belimumab), and prophylaxis for *Pneumocystis* pneumonia (trimethoprim-sulfamethoxazole and atovaquone). We considered the presence of renal involvement when including the four elements of SLEDAI, which are scores for the urinary cast, hematuria, proteinuria, and pyuria, serving as surrogate markers for nephritis. All patient information, including medication details, disease activity, and laboratory data such as blood cell counts, serum creatinine, CH50, IgG, HbA1c, and anti-dsDNA antibody levels, were measured and collected at enrollment and subsequent yearly observation points.

Logistic regression analysis with generalized estimating equations (GEE) was used to deal with intra-individual correlations and to estimate odds ratios for the incidence of severe infection. An exchangeable correlation structure was employed in the GEE. Survival curves were depicted using the Kaplan–Meier method and compared using a log-rank test for the period prior to the first severe infection during observation. Univariate and multivariate analyses were performed using Cox proportional hazards to predict associations with HCQ, other medications, disease activity, and demographic factors during the follow-up period. Multiple imputations were used for missing values. *P*-values below 0.05 were considered statistically significant. All statistical analyses were performed using SPSS V.28.0 (IBM, New York, USA) and R V4.1.0 (Foundation for Statistical Computing, Vienna, Austria).

### Ethical approval and informed consent

The study was conducted in accordance with the Declaration of Helsinki and ethical guidelines for epidemiological research in Japan. The study was approved by the Institutional Review Board of Yokohama City University (B180400002 and B181100009) and the institutional review board or ethics committee of each participating hospital. All patients were informed of the study at the time of enrollment and signed a consent form.

## Results

### Demographic and systemic lupus erythematosus-related variables

Of the 1,543 patients enrolled in the LUNA registry by 2021, 925 patients at 10 facilities with at least one year of the observation period were included in the study. Patients who lacked follow up data were excluded from the study. The duration of observation varied for each patient according to time of enrollment, and the number of patients followed during each period was as follows: 68 patients for 5 years, 165 patients for 4 years, 49 patients for 3 years, 230 patients for 2 years, and 413 patients for 1 year.

Eighty-eight percent of the 925 patients were female, with a median age of 45 years (interquartile range (IQR) 35–57). The median SLEDAI score, indicating SLE disease activity, was 4 (IQR 2–8), and the median SDI, a score quantifying organ damage, was 1 (IQR 0–2), suggesting that approximately half of the patients had relatively low disease activity. The median prednisolone (PSL) equivalent dose of glucocorticoid was 5.5 mg/day (IQR 4.0–9.0). 267 of 923 patients (28.9%) were using HCQ. The median anti-dsDNA antibody level was 7.7 IU/mL (IQR 2.3–17.5); 62.1% of the patients were taking at least one immunosuppressive drug for SLE disease control. Approximately 1 in 5 patients were taking oral prophylaxis against *Pneumocystis* pneumonia. The patient demographics and clinical characteristics, such as disease activity, treatment status, and immunological data of SLE, are described in **Table 1**.

**Table 1:** Patient characteristics at the baseline.

Variable	(*n =* 925)
Sex, female number (%)	815 (88.1)
Age (years)^a^	45 (35–57)
Clinical findings	
SELENA-SLEDAI score^a^	4 (2–8)
SDI score^a^	1 (0–2)
Renal involvement (%)^b^	298 (33.6)
End-stage renal failure (%)	27 (3.0)
Glucocorticoid dose (mg/day of PSL equivalent)^a^	5.5 (4.0–9.0)
Drugs	
Immunosuppressants, number (%)^c^	575 (62.1)
Mycophenolate mofetil (%)	124 (13.4)
Tacrolimus (%)	305 (33)
Cyclosporine (%)	60 (6.5)
Azathioprine (%)	105 (11.4)
Mizoribine (%)	39 (4.2)
Methotrexate (%)	27 (2.9)
Belimumab (%)	14 (2.3)
Cyclophosphamide (%)	5 (0.5)
Rituximab (%)	3 (0.3)
Hydroxychloroquine, number (%)	267 (28.9)
PCP prophylaxis, number (%)	185 (20)
Trimethoprim-sulfamethoxazole (%)	174 (18.9)
Atovaquone (%)	11 (1.5)
Laboratory data	
White blood cells (/μL)^a^	5,600 (4,263–7,140)
Neutrophil (/μL)^a^	3,890 (2,690–5,423)
Lymphocyte (/μL)^a^	1,128 (667-1,462)
Creatinine (mg/dL)^a^	0.68 (0.59–0.82)
CH50 (U/dL)^a^	37 (30–45)
IgG (mg/dL)^a^	1,325 (1,069–1,629)
HbA1c (%)^a^	5.6 (5.4–5.9)
Anti-dsDNA antibodies (IU/mL)^a^	7.7 (2.3–17.5)

a Values are expressed as median (interquartile range).

b At least one of the following: urinary cast, proteinuria, hematuria, and pyuria of SELENA-SLEDAI score.

c At least one of the following: cyclophosphamide, mycophenolate mofetil, mizoribine, methotrexate, azathioprine, tacrolimus, cyclosporine, rituximab, or belimumab.

SELENA-SLEDAI, SELENA-Systemic Lupus Erythematosus Disease Activity Index; SDI, Systemic Lupus International Collaborating Clinics/American College of Rheumatology Damage Index; PSL, prednisolone; PCP, Pneumocystis pneumonia.

### Frequency and types of severe infections

During the 5-year follow-up period, 95 patients developed severe infections, with 110 cases in total. Twelve patients had multiple infections (two at 5 years of follow-up, two at 4 years, two at 3 years, five at 2 years, and one at 1 year). Pneumonia was the most common type of infection (32 cases, 29.1%), followed by urinary tract infections (30 cases, 27.2%) (**Table 2**). Unfortunately, the identification of causative microorganisms for infections was challenging in many cases. However, for pneumonia, we identified *Staphylococcus aureus* in 2 cases, *Mycobacterium tuberculosis*, *Klebsiella pneumoniae*, *Haemophilus influenzae*, *Streptococcus pneumoniae*, and Pseudomonas aeruginosa in 1 case each. We also confirmed the presence of *Cryptococcus* in one case of fungal infection. For urinary tract infections, *Escherichia coli* was identified in 9 cases, and *Klebsiella pneumoniae*, *Klebsiella oxytoca*, *Streptococcus agalactiae*, and *Enterococcus faecalis* were each identified in 1 case. There were two cases of sepsis; however, information on the causative disease was not collected.

**Table 2:** Severe infections in SLE patients during the follow up period.

Infection	Number (*n* =110)	%
Pneumonia	32	29.1
Urinary tract infection	30	27.2
Skin and soft tissue infection	17	15.5
Intra-abdominal infection	7	6.4
Gastroenteritis	6	5.5
Sepsis	2	1.8
Infectious arthritis	2	1.8
Upper respiratory tract infection	1	0.91
Other	13	11.8

SLE, systemic lupus erythematosus.

### Risk factors affecting severe infection incidence

We investigated the factors influencing the incidence of severe infections during the follow-up period. We used generalized estimating equations, after missing value completion by multiple imputations, and analyzed 1,990 person-years, of which 92 were severe infection cases. Age, sex, treatment, and SLEDAI score were examined as candidate risk factors, with the glucocorticoid dosage logarithm transformed as a variable. Age, glucocorticoid dosage, and immunosuppressive drug use were identified as statistically significant risk factors for serious infections (**Table 3**). HCQ tended to decrease the rate of severe infections, but this was not statistically significant (odds ratio, 0.590 [95% CI 0.329–1.058], *p* = 0.077). Sex and SLEDAI score were not statistically significant risk factors.

**Table 3:** Generalized estimating equation logistic regression model for the risk of severe infections.

	Odds ratio (95% CI)	*p* Value
Sex (female)	1.335 (0.615–2.897)	0.47
Age	1.043 (1.027–1.060)	< 0.001
Glucocorticoid^a^	1.968 (1.379–2.810)	< 0.001
Immunosuppressants^b^	1.561 (1.025–2.380)	0.038
SLEDAI	0.983 (0.935–1.034)	0.52
Hydroxychloroquine	0.590 (0.329–1.058)	0.077

a Prednisolone equivalent dose was log-transformed for statistical analysis.

b At least one of the following: cyclophosphamide, mycophenolate mofetil, mizoribine, methotrexate, azathioprine, tacrolimus, cyclosporine, rituximab, belimumab.

CI, confidence interval; SLEDAI, Systemic Lupus Erythematosus Disease Activity Index.

Based on the finding that glucocorticoid dosage and immunosuppressant use contributed to severe infections in the first analysis, we also performed a subgroup analysis stratified by these factors. As the median steroid dose before logarithm transformation was 5 mg/day (IQR 3.0–7.0) in PSL equivalents, the patients were divided into two groups: those receiving ≤5 mg/day and those receiving >5 mg/day. The risk factors for serious infections in each group were compared. Age (odds ratio 1.041 [95% CI 1.019–1.063] vs 1.045 [1.020–1.070]) and log-transformed glucocorticoid dosage (2.007 [1.084–3.719] vs 2.047 [1.008–4.157]) contributed to the increase in severe infections in both PSL ≤5 mg/day and >5 mg/day groups, respectively. Immunosuppressant use was identified as a statistically significant risk factor only in the group with PSL dosage >5 mg/day (1.805 [1.014–3.211]). HCQ use suppressed severe infection in the >5 mg/day group more than in the ≤5 mg/day group, although the difference was not statistically significant (0.823 [0.351–1.928], *p* = 0.653 vs. 0.465 [0.211–1.030], *p* = 0.059).

We also divided the patients into two groups based on immunosuppressant use and compared their risk factors for severe infections. Similar to the two groups divided by glucocorticoid use, age and glucocorticoid dosage were identified as factors increasing severe infections in both the immunosuppressant use and non-use groups. HCQ use contributed to a decrease in severe infections in both the immunosuppressant-using and non-using groups, but this was not statistically significant. Furthermore, considering the potential effect on severe infections based on the types of immunosuppressants used, we performed a similar analysis with rituximab and cyclophosphamide as independent variables from the other immunosuppressants. The results showed that rituximab was found to contribute to the increase in severe infections (16.680 [4.761-58.446], *p* < 0.001) (**Supplementary Table 1**).

We tabulated the frequency of occurrence of each type of infectious disease in the HCQ and non-HCQ groups. The incidence of pneumonia was 3/520 cases/person-year in the HCQ group versus 27/1,470 cases/person-year in the non-HCQ group. Urinary tract infections occurred at a rate of 1/520 cases/person-year in the HCQ group versus 21/1,470 cases/person-year in the non-HCQ group. The incidence of these infections tended to be lower in the HCQ group compared to the non-HCQ group.

### Survival analysis for severe infection

**Figure 1:**
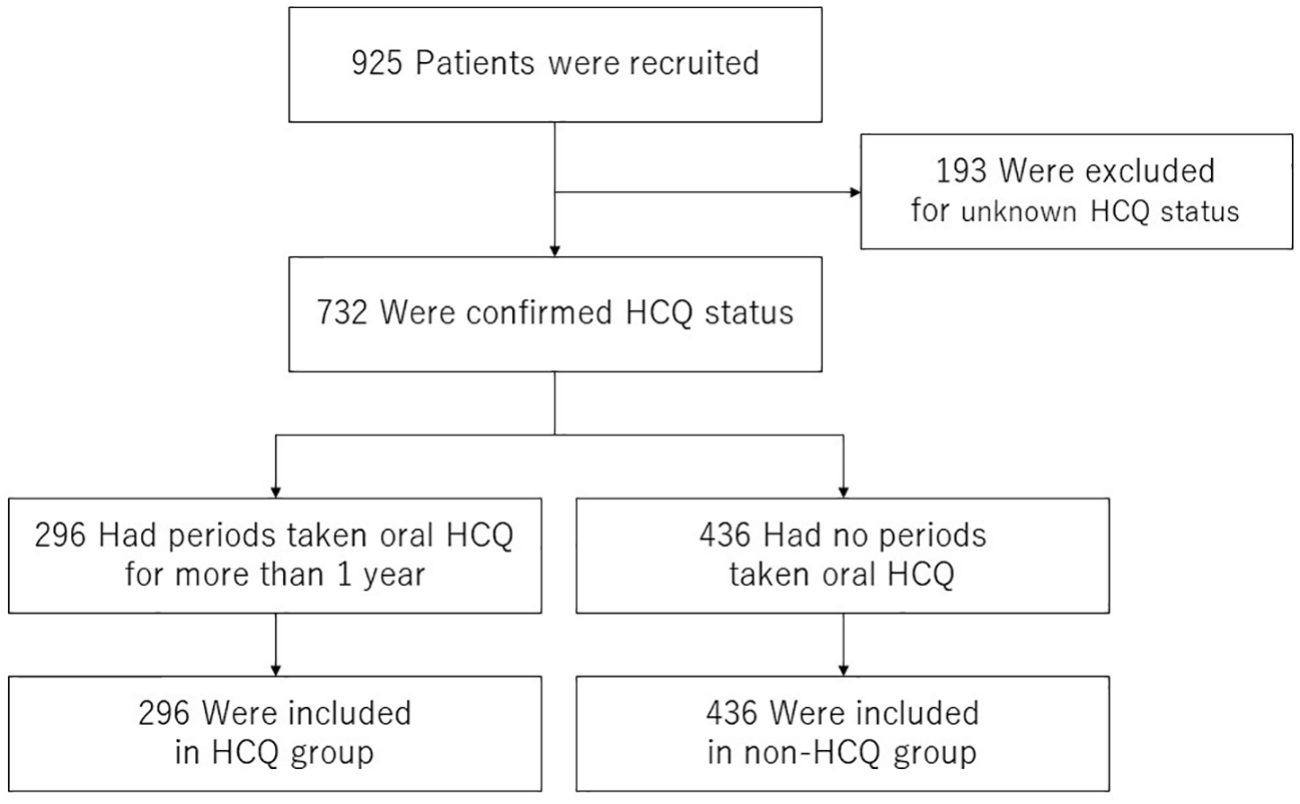
Patient selection flow chart. Of the 1,543 patients enrolled in the LUNA registry from 2016 to 2021, data from 925 patients who were followed up for > 1 year were included. A total of 193 patients were excluded because their HCQ adherence status during the follow-up was unknown. Of the remaining 732 patients, 296 who had been taking HCQ for more than one year were classified into the HCQ group and 436 who had never taken HCQ were classified into the non-HCQ group. HCQ, hydroxychloroquine.

To determine whether HCQ administration alters the incidence of severe infections, we performed survival analysis. We examined HCQ use in 925 registered patients who were followed up for at least one year. Patients confirmed to be taking HCQ at least two concurrent observation points were included in the HCQ group, and those who did not take HCQ during the follow-up period were included in the non-HCQ group. The 193 patients who could not be followed up were excluded from the analysis. Consequently, 296 and 436 patients were included in the HCQ and non-HCQ groups, respectively (**Figure 1**).

The baseline patient characteristics are shown in **Table 4**. Patients in the HCQ group were significantly younger than those in the non-HCQ group and had higher SLEDAI scores and anti-dsDNA antibody titers, indicating disease activity. The HCQ group had a significantly higher frequency of hypo-IgG than the non-HCQ group. There were also differences in treatment between the two groups, with glucocorticoid dosages and the rate of concomitant immunosuppressants being significantly higher in the HCQ group than in the non-HCQ group.

**Table 4:** Baseline patient characteristics in the survival analysis for first severe infection.

Variable	HCQ group (*n* = 296)	Non-HCQ group (*n* = 436)	*p* Value
Sex, female number (%)	269 (90.9)	395 (90.6)	1.0
Age (years)^a^	40 (32–49)	48 (40–62)	< 0.001
Clinical findings			
SELENA-SLEDAI score^a^	5 (2–8)	4 (2–6)	< 0.001
SDI score^a^	1 (0–1)	1 (0–2)	0.16
Renal findings^b^			0.21
No renal involvement (%)	179 (62.8)	276 (65.4)	
Renal involvement (%)	100 (35.1)	129 (30.6)	
End-stage renal failure (%)	6 (2.1)	17 (4.0)	
Glucocorticoid dose^a,c^	2.0 (1.8–2.4)	1.8 (1.4–2.2)	< 0.001
Drugs			
Immunosuppressants, number (%)^d^	208 (70.3)	236 (54.4)	< 0.001
Mycophenolate mofetil (%)	57 (19.3)	47 (10.8)	1.8×10^-3^
Tacrolimus (%)	113 (38.2)	121 (27.8)	3.9×10^-3^
Cyclosporine (%)	16 (5.4)	30 (6.9)	0.51
Azathioprine (%)	31 (10.5)	53 (12.2)	0.56
Mizoribine (%)	6 (2.0)	19 (4.4)	0.13
Methotrexate (%)	9 (3.0)	14 (3.2)	1.0
Belimumab (%)	8 (3.3)	1 (0.5)	0.039
Cyclophosphamide (%)	1 (0.3)	2 (0.5)	1.0
Rituximab (%)	3 (1.0)	1 (0.2)	0.31
PCP prophylaxis, number (%)	75 (25.3)	79 (18.1)	0.024
Trimethoprim-sulfamethoxazole (%)	70 (23.6)	75 (17.3)	0.043
Atovaquone (%)	5 (2.0)	4 (1.2)	0.51
Laboratory data			
White blood cells (/μL)^a^	5,475 (4,278–7,000)	5,600 (4,290–7,095)	0.93
Neutrophils (/μL)^a^	3,946 (2,735–5,509)	3,824 (2,683–5,275)	0.34
Lymphocyte (/μL)^a^	977 (662-1,427)	1,058 (688-1,554)	0.14
Creatinine (mg/dL)^a^	0.68 (0.59–0.79)	0.69 (0.59–0.84)	0.50
CH50 (U/dL)^a^	38 (30–48)	37 (30–45)	0.36
IgG (mg/dL)^a^	1,280 (996–1,535)	1,360 (1,091–1,717)	8.9×10^-3^
Hypo-IgG (%)^e^	29 (13.8)	21 (6.9)	0.015
HbA1c (%)^a^	5.6 (5.3–5.9)	5.7 (5.4–5.9)	0.077
Anti-dsDNA antibodies (IU/mL)^a^	10 (3.8–25.2)	5.9 (1.7–12.1)	< 0.001

^a^Values are expressed as median (interquartile range).

^b^At least one of the following: urinary cast, proteinuria, hematuria, and pyuria of SELENA-SLEDAI score.

^c^Prednisolone equivalent dose was log-transformed for statistical analysis.

^d^At least one of the following: cyclophosphamide, mycophenolate mofetil, mizoribine, methotrexate, azathioprine, tacrolimus, cyclosporine, rituximab, or belimumab.

^e^The lower reference limit for IgG was set at 870 mg/dL, and hypo-IgG was defined as lower than that value.

HCQ, hydroxychloroquine; SELENA-SLEDAI, SELENA-Systemic Lupus Erythematosus Disease Activity Index; SDI, Systemic Lupus International Collaborating Clinics/American College of Rheumatology Damage Index; PCP, Pneumocystis pneumonia.

**Figure 2:**
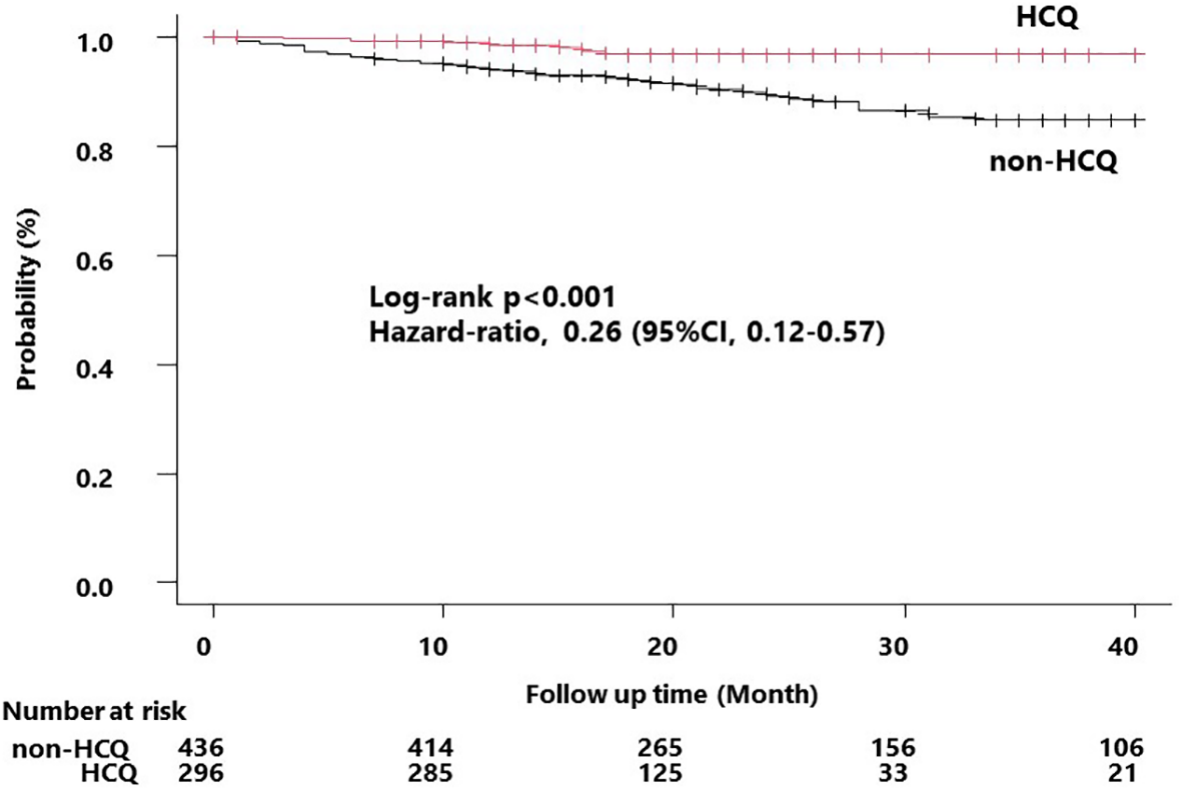
Kaplan–Meier curve for the occurrence of the first severe infection. Survival time curves for the HCQ and non-HCQ groups for the first severe infection that occurred during the follow-up period. The non-HCQ group had a significantly higher incidence of infection compared to that of the HCQ group. The hazard values were derived based on the univariate analysis. HCQ, hydroxychloroquine.

Using these patient data, we compared the incidence of severe infectious events in the HCQ and non-HCQ groups using univariate analysis. The median observation period was 16 and 24 months in the HCQ and non-HCQ groups, respectively. In all, 7 patients (2.4%) in the HCQ group and 50 (11.5%) in the non-HCQ group developed severe infections. The HCQ group had a lower incidence of severe infections than the non-HCQ group (*p* < 0.001, log-rank test; **Figure 2**). The severe infection-free rates at 24 months were 96.7% in the HCQ group and 89.1% in the non-HCQ group.

After multiple imputations to supplement missing values, we performed Cox regression analysis of factors associated with the incidence of severe infections during the follow-up period. Univariate analysis revealed that age at baseline and HCQ use were associated with the incidence of severe infections (hazard ratio (HR) 1.036 [1.019–1.054], *p* < 0.001 and 0.257 [0.116–0.570], *p* < 0.001, respectively) (**Table 5**). Multivariate analysis using the Cox proportional hazards model with sex, baseline age, and HCQ, which were significantly different in the univariate analysis, and glucocorticoid dosage and immunosuppressant use, which were identified as risk factors for severe infections in the GEE analysis, as covariates revealed that HCQ was significantly associated with the incidence of severe infections (HR 0.32 [0.142–0.728], *p* = 0.006).

**Table 5:** Cox proportional hazard analysis of the first severe infection in follow-up period.

Variable	Univariate analysis		Multivariate analysis	
	HR (95%CI)	*p* Value	HR (95%CI)	*p* Value
Sex (female)	0.601 (0.284–1,269)	0.18	0.828 (0.376–1.825)	0.64
Age	1.036 (1.019–1.054)	< 0.001	1.029 (1.009–1.050)	0.005
Glucocorticoid^a^	1.016 (0.985–1.049)	0.32	1,021 (0.993–1.049)	0.15
SLEDAI	1.039 (0.985–1.097)	0.16		
SDI	1.208 (1.037–1.406)	0.015	1.093 (0.915–1.306)	0.33
Immunosuppressants^b^	1.001 (0.582–1.722)	1.0	1.388 (0.788–2.445)	0.26
PCP prophylaxis^c^	1.209 (0.651–2.247)	0.55		
Hypo-IgG^d^	1.122 (0.345-3.651)	0.85		
Anti-dsDNA antibodies	0.999 (0.991–1.007)	0.84		
Hydroxychloroquine	0.257 (0.116–0.570)	< 0.001	0.322 (0.142–0.728)	0.006

^a^Prednisolone equivalent dose was log-transformed for statistical analysis.

^b^At least one of the following: cyclophosphamide, mycophenolate mofetil, mizoribine, methotrexate, azathioprine, tacrolimus, cyclosporine, rituximab, belimumab.

^c^At least one of the following: trimethoprim-sulfamethoxazole, atovaquone

^d^The lower reference limit for IgG was set at 870 mg/dL, and hypo-IgG was defined as lower than that value.

HR, hazard ratio; CI, confidence interval; SELENA-SLEDAI, SELENA-Systemic Lupus Erythematosus Disease Activity Index; SDI, Systemic Lupus International Collaborating Clinics/American College of Rheumatology Damage Index; PCP, Pneumocystis pneumonia.

## Discussion

In recent years, mortality in patients with SLE has gradually declined due to changes in treatment strategies (16). Lorenzo-Vizcaya et al. analyzed trends in the causes of death in patients with SLE over the past 40 years and reported that infectious diseases have overtaken cardiovascular disease as the most common (3). This suggests that the control of infectious disease is important in improving the prognosis of patients with SLE. The use of HCQ, an antimalarial drug, has become the global standard for the treatment of SLE because of its disease suppression activity. Several reports have shown that HCQ significantly limits infectious disease complications (8–11). In this study, we evaluated the efficacy of HCQ in reducing severe infections using a multicenter SLE registry of Japanese data. First, we examined the association between HCQ and severe infections using GEE and found a non-significant relationship between HCQ use and a reduction in severe infections. Second, using survival time analysis we found that HCQ significantly reduced severe infections.

In Japan, HCQ was approved in 2015; this was done later than in other countries due to a pharmacotoxicity incident caused by chloroquine, resulting in limited HCQ data in Japanese patients. There are still many patients with SLE in Japan who do not use HCQ, providing a suitable population for analyzing its effects. In our study, 28.9% of the patients were using HCQ at enrollment; this may be because the cohort started in 2016, and a quarter of the patients were enrolled in the first two years. Approximately 40% of patients in the survival time analysis were taking HCQ, suggesting that usage gradually increased during the observation period. There were no patients whose actual HCQ use was higher than the dosage specified in the range of ±10% of ideal body weight, and only 7.7% of all patients used less than the specified dosage.

The HCQ group in our study showed a younger age and higher disease activity compared to the non-HCQ group. This can be attributed to the recent approval of HCQ in Japan and the proactive trend of introducing it in newly diagnosed or relapsed SLE patients. HCQ has been recommended by various societies to be used in all patients with SLE unless there are contraindications. However, patients who have remained in remission and stable since before the HCQ was approved often do not dare to introduce the HCQ. Such historical background is thought to be one of the reasons for the lower population age and the higher disease activity of patients using HCQ. Furthermore, the HCQ group had significantly lower serum IgG levels and a higher percentage of patients with serum IgG levels below the reference range than the non-HCQ group. This may be due in part to the higher dose of glucocorticoids and higher use of immunosuppressive agents in the HCQ group compared to the non-HCQ group.

In this study, the incidence of severe infections during the follow-up period was 5.47 infections/100 person-years, which may have been influenced by regional and racial differences. A total of 110 infections were observed during the follow-up period, including pneumonia, urinary tract infections, and skin and soft tissue infections, similar to those reported previously (9, 17, 18). Although PCP should be considered when glucocorticoids or immunosuppressive agents are used in the treatment of SLE, the incidence of PCP is unknown because not all data on the causative pathogen of pneumonia were collected in this registry.

Various reports have shown that HCQ reduces the risk of mortality in SLE (19), but few have identified the suppression of infections by HCQ as a mechanism. Ruiz-Irastorza et al. showed an inverse association between antimalarial drug use and infection (OR 0.06 [95%CI 0.02–0.18]) in a cohort study of 249 subjects in 2009 (8). The Spanish researchers also observed that an increased duration of antimalarial drug use is protective against infection, using a group of 3,658 patients in 2017 (9). However, the protective effects do not appear to be clinically significant (HR 0.998 [0.997–0.999] 95% CI). Recently, a study of a multinational Latin American cohort with 1,243 SLE patients showed that antimalarials have a protective effect against serious infection (HR 0.69 [0.48–0.99]) (20). However, these studies were dominated by Caucasian and mestizo populations, with few Asian participants, and the age at enrollment was 9–18 years younger on average than that in our study.

In Asia, a Chinese group examined the association between antimalarials and cause-specific mortality in patients with SLE (2). They concluded that antimalarials were associated with lower overall mortality from SLE and, in a cause-specific analysis, were inversely associated with death from renal failure and other causes. There was no significant association with death from infectious diseases, although the study did show a trend toward lower mortality from infectious diseases among antimalarial drug users (adjusted HR 0.79 [0.49–1.29]). However, because the data represented infectious disease mortality, not incidence, and the participants had a high disease burden, the association between HCQ and the incidence of infectious diseases itself remained unclear. In view of these reports, it is debatable whether HCQ is effective in preventing infectious diseases.

In this study, we used GEE with the person-year method to analyze longitudinal data. Using data from a one-year follow-up study, we were able to analyze the short-term effect of HCQ in reducing infections in GEE with real-life data. We identified age, glucocorticoid use, and immunosuppressant use as risk factors for severe infections, similarly to previous reports (17). We could not identify HCQ use as a significant factor associated with serious infections, but we observed a tendency to suppress infection with an HR of 0.59 [0.329–1.058].

Furthermore, survival analysis of the effect of HCQ on severe infections showed that the HCQ group had a significantly lower incidence of infections than the non-HCQ group. Survival time analysis confirmed the long-term effects of HCQ on suppressing infections. Although the HCQ group had a 7.6% lower incidence of infections during the 2-year observation period compared to the non-HCQ group, at baseline, the HCQ group had higher anti-dsDNA antibody titers and SLEDAI scores, higher glucocorticoid dose levels and concomitant immunosuppressant usage compared with those of the non-HCQ group. As the HCQ group had a higher risk of infectious complications, HCQ might have an even greater inhibitory effect on infectious diseases. The survival analysis showed a similar trend to the GEE analysis but revealed a clear and significant difference in the effect of HCQ on the suppression of infectious diseases. This suggests that the duration of administration or cumulative exposure may be important for the effect of HCQ in preventing infectious diseases; further investigation is warranted.

The GEE analysis also showed that glucocorticoid dosage and age were risk factors for severe infections. It is relatively easy to infer that older age and higher glucocorticoid doses contribute to infection. Because PSL has been reported to contribute to infection in patients with SLE (21, 22) and long-term glucocorticoid use leads to irreversible organ damage (23, 24), it is important to reduce the PSL dose with concomitant immunosuppressive drugs (7). It has been reported that the PSL dose can be reduced by concomitant use of HCQ (25), and we believe that HCQ can more effectively limit infections, both when compared to glucocorticoids and in its own right.

HCQ was initially used as an antimalarial drug and is currently used in autoimmune diseases such as SLE and RA owing to its anti-inflammatory and immunomodulatory effects. It has been reported to inhibit vesicular enzyme function and antigen presentation to MHC class II through the accumulation of HCQ in lysosomes, inhibition of the associated production of inflammation-inducing cytokines such as IL-1, IFNα, and TNF, and inhibition of the Toll-like receptor signaling pathways such as TLR7 and TLR9 (26, 27). Several reports have speculated on the mechanism by which HCQ exerts its antimicrobial activity. HCQ inhibits the growth of intracellular bacteria, mainly by alkalinizing phagosomes (28), and fungal growth by limiting the release of iron in a pH-dependent manner in phagolysosomes (29). For viruses, HCQ prevents entry into host cells via endocytosis by increasing the pH of lysosomes and inhibiting the post-translational modification of glycoproteins in the viral envelope (30). Recently, some reports have suggested that HCQ is also effective against COVID-19 infections by inhibiting the binding of angiotensin-converting enzyme-2 (ACE-2) and ganglioside to the SARS-CoV-2 spike protein (31). However, some of the previously reported studies were *in vitro*, and many aspects of the role of HCQ in inhibiting infections remain unknown, requiring further study.

This study had some limitations. Because this was a retrospective study and not all clinical data were collected over time, there were some missing data. Although HCQ requires approximately 4 months to reach a steady blood concentration and its half-life is 40 to 50 days (32, 33), this study did not consider medication adherence and the period between the initiation or discontinuation of oral drug administration and the cross-sectional observation point. Although previous reports have suggested that the duration and cumulative dosage of HCQ may be associated with the suppressive effect on infection (9, 10), our study did not collect data on HCQ oral status or dosage before enrollment, thereby making these analyses difficult. We were also unable to analyze the dose-dependent effect of HCQ on infections because most HCQ-using patients were prescribed exactly the dose determined by their ideal body weight. In addition, the reason for HCQ prescription was not adjusted in this study.

Furthermore, the limited number of severe infections in this analysis made it difficult to statistically examine the effect of each immunosuppressant individually. However, since the effect of each immunosuppressant on infectious diseases is expected to differ for each individual immunosuppressant, we will plan to examine the effect of individual immunosuppressants after accumulating more cases. The cohort mainly consisted of patients followed up in the rheumatology departments of large medical facilities in Japan and did not include patients who did not visit the rheumatology departments but only the dermatology departments. In Japan, some rheumatologists with a background in dermatology may independently follow up patients with SLE whose main symptom is a skin rash without vital organ involvement, and it is well known that the HCQ is effective in treating skin lesions in SLE (34, 35). Therefore, it is possible that some patients with mild SLE may have been overlooked, and this may have influenced the results of this study. However, since the majority of patients with SLE are considered to be attending rheumatology clinics, we do not believe that this influence is significant.

In addition, since hospitals in Japan have different roles and treat different diseases depending on their size, it is necessary to consider the possibility that common diseases, such as pneumonia and urinary tract infections, were treated at other hospitals excluded from the database. The ethnicity of the patients in this cohort was not recorded; however, foreign residents accounted for only 1.88% of Japan’s total population in 2016, of which 58.4% were from three East Asian countries: China, Korea, and the Philippines (36). Given the social environment in Japan, it is assumed that most people are of Japanese descent. Another strength of this study is that, unlike other studies, it compared the effects of HCQ in a homogeneous population.

In conclusion, we have demonstrated the data suggesting that HCQ can have the potential to inhibit infections. Infection is a potentially fatal SLE co-morbidity and can interfere with treatment. As HCQ not only reduces disease activity in SLE but also inhibits the development of infections, its role in the treatment of SLE is essential. Further prospective studies are required to investigate this further.

## Data availability statement

The raw data supporting the conclusions of this article will be made available by the authors, without undue reservation.

## Ethics statement

The studies involving humans were approved by the Institutional Review Board of Yokohama City University. The studies were conducted in accordance with the local legislation and institutional requirements. The participants provided their written informed consent to participate in this study.

## Author contributions

Conception and design: RY. Data analysis and interpretation, drafting and editing of manuscripts: CH, RY, and HNak. Statistical analysis: YSai and JT. Patient follow-up, analysis and interpretation of data: NK, NSu, NSa, YY, YS-K, YKu, DK, KH, YSat, TK, HNag, NH, AM, NT, LH, YSo, KT-M, YKi, NY, K-ES, YM, KI, SO, HK, SS, YSh, and MF. Critical review of papers and approval of manuscripts: all authors.
