# Supplementary material for: Protective effect of hydroxychloroquine on infections in patients with systemic lupus erythematosus: an observational study using the LUNA registry
Source: Front Immunol. 2023 Sep 1;14:1227403. doi: 10.3389/fimmu.2023.1227403 (PMC10504661; doi:10.3389/fimmu.2023.1227403)
Supplement: Supplementary file 1 [file Table_1.pdf]

Supplemental Table 1 Generalized estimating equation logistic regression model for the risk of severe infections with cyclophosphamide and rituximab as independent variables.

	**Odds ratio (95% CI)**	***p* Value**
Sex (female)	1.271 (0.584-2.768)	0.55
Age	1.044 (1.028-1.061)	< 0.001
Glucocorticoid[a]	1.896 (1.334-2.694)	< 0.001
Cyclophosphamide	3.160 (0.505-19.788)	0.22
Rituximab	16.680 (4.761-58.446)	< 0.001
Other immunosuppressants[b]	1.359 (0.854-2.163)	0.20
SLEDAI	0.985 (0.938-1.035)	0.56
Hydroxychloroquine	0.606 (0.335-1.099)	0.099

[a]Prednisolone equivalent dose was log-transformed for statistical analysis.

[b]At least one of the following: mycophenolate mofetil, mizoribine, methotrexate, azathioprine, tacrolimus, cyclosporine, belimumab.

CI, confidence interval; *SLEDAI*, Systemic Lupus Erythematosus Disease Activity Index.
